# Maternal obesity and fetal deaths: results from the Brazilian cross-sectional demographic health survey, 2006

**DOI:** 10.1186/1471-2393-14-5

**Published:** 2014-01-07

**Authors:** Mariana Santos Felisbino-Mendes, Fernanda Penido Matozinhos, J Jaime Miranda, Eduardo Villamor, Gustavo Velasquez-Melendez

**Affiliations:** 1 Department of Maternal and Child Nursing and Public Health, Escola de Enfermagem, Universidade Federal de Minas Gerais (UFMG), Av. Alfredo Balena, 190 - Bairro Santa Efigênia, 30130-100, Belo Horizonte, MG, Brasil; 2 CRONICAS Center of Excellence in Chronic Diseases, and Department of Medicine, School of Medicine, Universidad Peruana Cayetano Heredia, Lima, Peru; 3 Department of Epidemiology, School of Public Health, University of Michigan, Ann Arbor, MI, USA

**Keywords:** Spontaneous abortion, Stillbirth, Obesity, Body mass index, Waist circumference

## Abstract

**Background:**

Obesity is highly related to negative reproductive health outcomes, but its relationship with spontaneous abortion and stillbirth remains to be understood, especially in transitioning economies. This study aimed to examine the relationship between obesity and spontaneous abortions and stillbirths in a representative sample of the Brazilian population.

**Methods:**

Cross-sectional study using secondary data of Brazilian women of reproductive age (15–45 years old) from the National Demographic and Health Survey in 2006. Obesity was measured by body mass index (BMI), waist circumference (WC) and waist-to-height ratio (WHR). Logistic regression modeling of the survey data was used to evaluate the relationship between obesity and the study outcomes.

**Results:**

The three obesity markers used were found to be strongly and positively associated with spontaneous abortion and stillbirth occurrence. In the adjusted models, there was strong evidence that for each unit increase in BMI (OR = 1.05; 95%CI: 1.02-1.08) and WHR (OR = 1.32; 95%CI: 1.03-1.69), the odds of having a spontaneous abortion was higher. In addition, compared to those of optimal weight, obese women were more likely to have negative outcomes. Maternal age, parity, skin color, educational level and household income were important covariates for adjustment. A sensitivity analysis among women who had only one pregnancy was also performed and showed similar results.

**Conclusion:**

Obesity is potentially associated with an increased risk of spontaneous abortion and stillbirth in a representative sample of the Brazilian population. These findings are in accordance with previous studies and thus reinforce the need for obstetric care providers to counsel obese reproductive-age women regarding the risks, complications and importance of weight loss and weight control prior to pregnancy.

## Background

Obesity is increasing worldwide and has significant health-related consequences, including increased mortality [1,2]. Obesity is a growing problem, particularly among women, who have a combined prevalence of overweight and obesity of over 20% [3-5].

This increase is also observed in middle-income countries, such as Brazil, a country that is rapidly transitioning with an emerging economy. For instance, national surveys in the last 34 years have shown a continuous increase in overweight and obesity prevalence among Brazilian men and women. Most up-to-date estimates show that overweight and obesity levels are approximately 50% and 15%, respectively [6]. In reproductive-age women, the prevalence of obesity is approximately 17%.

Being overweight is a significant handicap in women undergoing infertility treatment, and women are often encouraged to lose weight before treatment [2,7]. Additionally, maternal obesity is associated with various risks in pregnancy and to offspring [7]. It leads to changes in maternal metabolism, and some studies have also associated obesity with abortion, late fetal death and infertility risk [2,8,9]. Moreover, consequences of maternal obesity may extend beyond fetal life into childhood and adulthood, when the fetus becomes a viable and live birth [2,10,11]. This relationship has been consistently shown in studies in high-income countries, but poorly explored in low and middle-income nations, such as Brazil [12-14], where obesity is a rapidly growing problem for public health.

Most of the previous research regarding the association between obesity and reproductive outcomes only used body mass index (BMI) as an obesity marker. On the other hand, waist circumference (WC), a more direct measure of the abdominal accumulation of fat [15], and waist-to-height ratio (WHR), a more practical index for screening the risk of obesity-related disorders that could be applied uniformly to all ethnicities and ages [16], are also important and reliable obesity indexes [16-18]. This study aimed to examine the potential relationship between obesity and the occurrence of spontaneous abortion and stillbirths in a representative sample of the Brazilian female population.

## Methods

### Data sources, study population and sample size

The data for this study came from the 2006–2007 Brazilian National Demographic and Health Survey (DHS), which is a nationally representative cross-sectional study carried out in 2006 by the Ministry of Health designed to evaluate maternal and child health. Data collection was performed through home interviews and exams of non-institutionalized women at reproductive age (15 to 49 years). Detailed sampling plans, data collection information and data quality assurance are available in the DHS 2006 survey final reports at http://bvsms.saude.gov.br/bvs/pnds/index.php.

**Figure 1:**
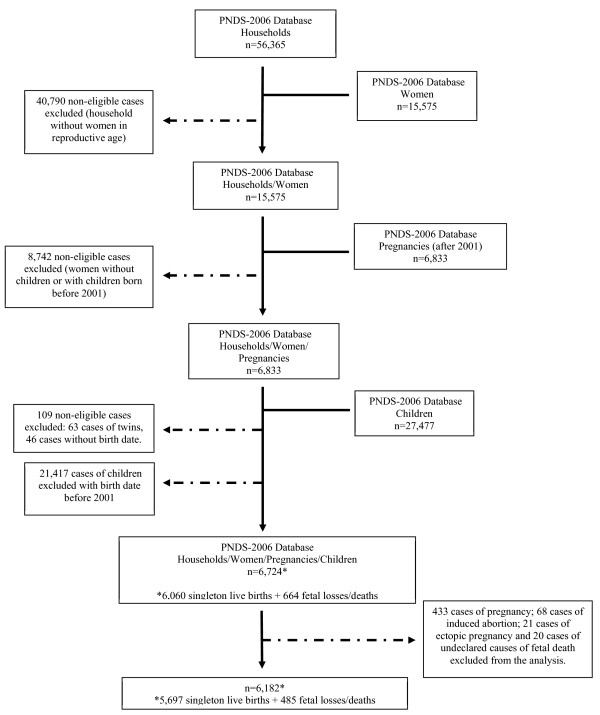
Study population flowchart.

The DHS 2006 sample was stratified by urban and rural areas and by geographic region: Northeast, Southeast, North, Mid-West and South. The sample was composed of 14,617 households with 56,365 women; 15,575 of them were of reproductive age and 6,833 had reported at least one pregnancy after 2001. From this number, 109 women were excluded due to twin births or absence of data regarding the event (birth or death). Thus, from the 6,724 women available for the study, 11.6% (SE 0.7) declared fetal deaths. Of these deaths, 70.2% (SE 3.0) were spontaneous abortions and 11.4% (SE 1.9) were stillbirths. Other types of deaths, such as ectopic pregnancy and induced abortion, were not considered in this study (18.4%; SE 2.6) (n = 109). Pregnant women were also excluded from the sample (n = 433). The total sample subpopulation was 6,182 (Figure 1).

### Outcome, exposure of interest and confounders

The endpoint for our study was fetal deaths, derived from the pregnancies that occurred from 2001 until the time of the interview. This general outcome included spontaneous abortions (≤ 20 weeks of gestational age) and stillbirths (> 20 weeks of pregnancy). Both of them were self-reported and binary (1 = abortion/stillbirth, 0 = otherwise).

Obesity was the exposure of interest, and it was ascertained through three different indicators: WC, WHR and BMI. The waist circumference categories were determined as follows: normal (< 80 cm), overweight (80–87.9 cm) and obesity (≥ 88 cm). WHR was calculated dividing the women’s WC by their height, both measured in centimeters, and the categories were defined by quartiles of distribution, where the first quartile consisted of the lowest values and the fourth consisted of the highest. Height and weight were used to calculate BMI according to the formula weight/height^2^, and the women were classified into the following categories: undernutrition (< 18.5 kg/m^2^), eutrophic (18.5-24.9 kg/m^2^), overweight (25.0-29.9 kg/m^2^), obesity class I (30.0-34.9 kg/m^2^) and obesity class II-III (≥ 35.0 kg/m^2^). All indexes were analyzed as continuous and categorical variables, and WC and BMI were classified according to conventional cut-off points [1,19].

**Figure 2:**
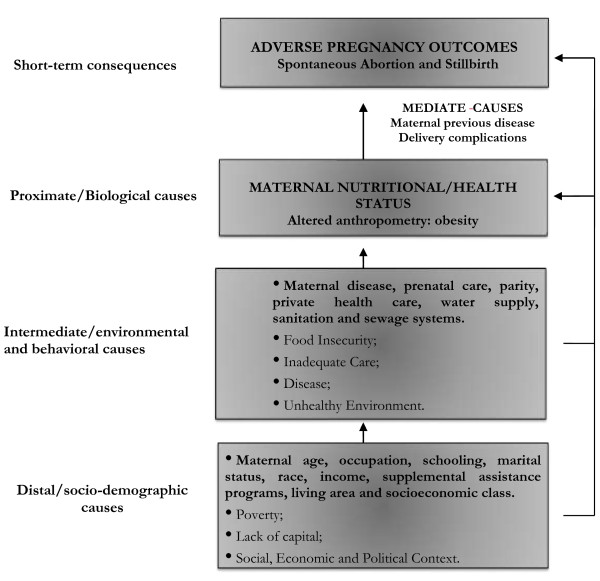
**Framework of the relationship between the causes of maternal obesity and the fetal consequences.** Adapted from Black et al., 2008 and Mosley & Chen, 1984.

The potential confounders were considered according to the conceptual model (Figure 2) [20,21], and important adjustments were made when related to the outcome and the exposure of interest, such as maternal age group (15–19; 20–24; 25–29; 30–34; 35–39; ≥ 40), educational level in years (illiteracy; 1–4; 5–8; 9–11; 12 or more years), skin color as a proxy for race and socioeconomic status (white, brown, black, others), parity (0–1; 2–3; ≥ 4 children) marital status (single; married or cohabitating; widowed; divorced), occupation (yes or no), smoking status (yes or no), and quartiles of household income (1st quartile: 0–270, 2nd quartile: 271–500, 3rd quartile: 501–972 and 4th quartile: ≥ 973; in 2006–2007, approximately US$ 0.47 dollars per Brazilian *real*) and area of residence (urban/rural).

### Analysis

Analyses were performed with STATA version 12.0 (Stata Corp., College Station, TX, USA), using survey commands, to account for the complex sample survey data composition: strata, clusters and weights. Instead of excluding participants, we conducted an unconditional subpopulation analysis [22], restricting the estimate to a subpopulation of interest. This type of performance is highly recommended when analyzing complex designed data because it is a more appropriate approach to variance estimates [22]. Out of a total sample of 6,724 participants, our subpopulation included 6,182 non-pregnant women with a history of pregnancy after 2001 to data collection (single live births or incomplete pregnancies) (Figure 1). Thus, we treated the exclusion conditions as category 0 of the subpopulation indicator and the population of interest as category 1 [22].

Proportions and standard errors (SE) were estimated by descriptive analysis. We also estimated unadjusted and adjusted odds ratios (OR) and 95% confidence intervals (95% CI) for all outcomes according to obesity markers. The logistic regression models were adjusted by maternal age group, parity, skin color, household income, maternal education and maternal smoking status. Wald design-based chi-square and goodness of fit tests were also performed to evaluate the fitted models [23].

Finally, we conducted a sensitivity analysis that consisted of the same performance but restricted to the women who reported only one pregnancy at the time of the interview. This analysis consisted in an effort to attenuate potential effect of changes, usually increases, in obesity indicators in multiparous women.

### Ethical review

Our study used de-identified data. The DHS was conducted following standard ethical guidelines established by Helsinki Declaration and was approved by the Ethical Committee Council of the State Health Secretary of Sao Paulo.

## Results

### Sociodemographic characteristics

We examined the distribution of demographic and socioeconomic characteristics in the total population and according to the reproductive health outcomes studied (Table 1). The majority of women in the sample analyzed were < 40 years old (93%), living in urban areas (80%) and had a higher educational level with 9 or more years of education (43%). Of all participants, 51% had 2 or 3 children and approximately 85% of the women were married.

**Table 1:** Demographic and socioeconomic characteristic distribution in the total study population and according to reproductive outcome – DHS/2006, Brazil

**Characteristics**	**Total population**		**Spontaneous abortion**	**Stillbirth**	**All deaths**^**a**^
	**n**^**b**^	**% ± (SE)**	**% ± (SE)**	**% ± (SE)**	**% ± (SE)**
**Age**					
15-19	448	8.8 (0.8)	8.4 (1.7)	2.4 (1.3)	10.6 (2.1)
20-24	1726	30.3 (1.2)	6.9 (1.2)	1.2 (0.5)	7.9 (1.2)
25-29	1733	25.4 (1.0)	5.6 (0.9)	1.4 (0.4)	6.9 (1.0)
30-34	1215	17.7 (1.0)	7.4 (1.3)	0.7 (0.3)	8.1 (1.3)
35-39	678	11.1 (0.8)	11.1 (2.4)	1.7 (0.9)	12.6 (2.5)
≥ 40	382	6.8 (0.7)	15.1 (3.2)	3.2 (2.0)	17.9 (3.5)
**Area of residence**					
Urban	4063	79.6 (1.6)	8.1 (0.8)	1.2 (0.3)	9.0 (1.2)
Rural	2119	20.5 (1.6)	6.8 (1.2)	2.4 (0.7)	9.2 (0.8)
**Schooling (years)**					
0	205	2.7 (0.4)	2.9 (2.0)	6.5 (4.2)	9.2 (4.4)
1-4	1424	19.0 (1.3)	6.0 (1.2)	1.7 (0.7)	7.6 (1.3)
5-8	2215	35.7 (1.4)	7.9 (1.0)	1.5 (0.4)	9.3 (1.1)
9-11	1911	35.4 (1.5)	8.1 (1.1)	0.7 (0.2)	8.7 (1.1)
12+	380	7.2 (0.8)	11.9 (2.7)	1.9 (1.4)	13.6 (3.1)
**Skin color**					
White	2093	33.9 (1.4)	6.9 (0.9)	1.3 (0.5)	8.1 (1.0)
Brown	3068	49.5 (1.6)	7.4 (1.0)	1.8 (0.4)	9.1 (1.0)
Black	620	11.0 (1.1)	11.5 (2.1)	0.9 (0.5)	12.3 (2.1)
Other	329	5.6 (0.7)	10.7 (3.3)	0.1 (0.1)	10.8 (3.3)
**Parity**					
0-1	1944	38.3 (1.4)	12.8 (1.4)	1.9 (0.5)	14.5 (1.4)
2-3	3070	51.2 (1.4)	4.9 (0.7)	1.1 (0.4)	6.0 (0.7)
≥ 4	1168	10.5 (0.6)	3.8 (1.1)	1.0 (0.4)	4.8 (1.1)
**Marital status**					
Single	337	5.5 (0.8)	9.0 (2.9)	2.4 (1.2)	11.1 (3.0)
Married	5157	85.2 (1.1)	7.8 (0.7)	1.5 (0.3)	9.1 (0.8)
Widowed	38	0.6 (0.2)	3.1 (2.6)	0.6 (0.6)	3.6 (2.8)
Divorced	646	8.7 (0.8)	8.0 (1.7)	0.4 (0.2)	8.3 (1.7)
**Smoking Status**					
Yes	1,000	16.3 (1.0)	10.2 (1.8)	1.8 (0.9)	11.8 (1.8)
No	5,182	86.7 (1.0)	7.4 (0.7)	1.4 (0.3)	8.6 (0.7)
**Income**^**c**^					
1st Quartile (0–270)	1325	22.0 (1.4)	6.1 (1.0)	2.2 (0.8)	8.2 (1.2)
2nd Quartile (271–500)	1521	26.4 (1.6)	6.4 (1.1)	2.2 (0.7)	8.5 (1.1)
3rd Quartile (501–972)	1187	21.6 (1.1)	8.3 (1.4)	1.3 (0.6)	9.5 (1.5)
4th Quartile (≥ 973)	1406	30.0 (1.7)	10.3 (1.7)	0.5 (0.3)	10.8 (1.7)

^a^Abortions and Stillbirths ^b^n = real sample values. ^c^Value in reais (in 2006–07, US$ 0.47 per real). *SE* Standard Error.

### Prevalence of abortion and stillbirths

Women of advanced age had a higher proportion of abortions and stillbirths. Stillbirths were more frequent at either very young or very old ages. The proportion of abortions also increased with maternal education, but an inverse relationship was observed for stillbirths. Furthermore, total fetal deaths were more frequent among women with a marital status of single, women who reported 0–1 children and women whose skin color was declared black and brown (Table 1).

The proportion of abortions and stillbirths were higher among overweight and obese women (Table 2). The proportion of fetal deaths was also higher in abdominally obese women (10.2%) and in women with the highest values of WHR (4th quartile, 10.9%). The unadjusted association between any index of obesity and total deaths was significant (Table 2). In these preliminary models, women with higher BMI values (≥ 35 Kg/m^2^) presented an almost twofold higher odds of abortion (OR = 1.96; 95%CI: 1.16-3.30) compared to those with lower values of this anthropometric marker. This relationship was not found for abdominal obesity (WC) or higher values of WHR, only after controlling for confounders.

**Table 2:** Proportions (SE) and unadjusted OR (95% CI) of spontaneous abortions and stillbirths according to maternal anthropometry – Brazil, DHS/2006

**Maternal anthropometry**	**n**	**Spontaneous abortions**		**Stillbirths**		**Total deaths**	
		**% ± (SE)**	**Unadjusted OR (95%CI)**	**% ± (SE)**	**Unadjusted OR (95%CI)**	**% ± (SE)**	**Unadjusted OR (95%CI)**
**Maternal BMI (Kg/m**^**2**^**)**							
Underweight (<18.5)	251	4.5 (1.7)	0.55 (0.25-1.22)	0.1 (0.1)	0.08 (0.01-0.63)^*^	4.6 (1.7)	0.49 (0.22-1.06)
Normal (18.5-24.9)	3,302	7.8 (0.8)	1.00	1.2 (0.3)	1.00	9.0 (0.8)	1.00
Overweight (25–29.9)	1,645	7.4 (1.3)	0.94 (0.63-1.41)	1.4 (0.5)	1.10 (0.44-2.79)	8.6 (1.3)	0.96 (0.67-1.56)
Obese 1 (≥ 30 -34.99)	625	6.6 (1.6)	0.84 (0.49-1.44)	2.2 (0.9)	1.83 (0.72-4.67)	8.7 (1.8)	0.97 (0.60-1.56)
Obese 2–3 (≥ 35)	359	14.3 (3.1)	1.96 (1.16-3.30)^*^	3.4 (2.4)	2.81 (0.60-13.22)	17.1 (2.8)	2.10 (1.36-3.25)^*^
**Maternal BMI (per 1 Kg/m**^**2**^**)**	**6,182**	-	1.03 (1.00-1.07)	-	1.07 (1.00-1.14)^*^	-	1.04 (1.01-1.07)^*^
**Maternal WC (cm)**							
Normal (< 80)	2,782	7.4 (0.8)	1.00	0.9 (0.3)	1.00	8.3 (0.9)	1.00
Overweight (80–87.9)	1,457	7.9 (1.2)	1.06 (0.71-1.60)	1.3 (0.6)	1.41 (0.48-4.23)	9.1 (1.3)	1.10 (0.75-1.60)
Obese (≥ 88)	1,758	8.2 (1.3)	1.10 (0.74-1.63)	2.4 (0.6)	2.61 (1.13-6.04)^*^	10.2 (1.3)	1.26 (0.90-1.75)
**Maternal WC (per 1 cm)**	**5,997**	-	1.01 (1.00-1.03)	-	1.03 (1.00-1.06)	-	1.02 (1.00-1.03)^*^
**Maternal WHR**							
1st quartile	1,494	6.6 (1.0)	1.00	0.8 (0.4)	1.00	7.4 (1.0)	1.00
2nd quartile	1,495	8.1 (1.3)	1.25 (0.78-1.98)	0.9 (0.4)	1.09 (0.30-3.92)	8.9 (1.3)	1.23 (0.80-1.90)
3rd quartile	1,490	8.0 (1.2)	1.23 (0.79-1.93)	1.3 (0.5)	1.53 (0.48-4.88)	9.2 (1.3)	1.27 (0.84-1.93)
4th quartile	1,494	8.4 (1.5)	1.30 (0.80-2.12)	2.8 (0.8)	3.42 (1.15-10.13)^*^	10.9 (1.5)	1.55 (1.02-2.35)^*^
**Maternal WHR (per 0.1)**	**5,973**	-	1.19 (0.94-1.51)	-	1.78 (1.13-2.81)^*^	-	1.29 (1.06-1.55)^*^

^*^p < 0.05; *BMI* Body Mass Index, *WC* Waist Circumference, *WHR* Waist-to-Height Ratio, *OR* Odds Ratio, *SE* Standard Error, *95%CI* 95% Confidence Interval.

### Association between obesity markers and outcomes

Women with WC values equal to or greater than 88 cm were also more likely to report a stillbirth (OR = 2.61; 95%CI: 1.13-6.04) when compared to those with lower and median values for these measures (p < 0.05). The same pattern was observed for those who had WHR values in the 4th quartile of the distribution (p < 0.05). These associations remained unchanged after adjusting for maternal age group, parity, skin color, educational level, smoking status and household income (Table 3). Adjusted OR and 95% confidence intervals for the associations between obesity markers and the reproductive outcomes showed that all anthropometric indexes positively associated with the total deaths. There was strong evidence that for each unit increase in BMI (OR = 1.05; 95%CI: 1.02-1.08) and WHR (OR = 1.32; 95%CI: 1.03-1.69), the odds of having a spontaneous abortion was higher. We also observed that abdominal obesity (WC ≥ 88 cm) was strongly associated with stillbirths (OR = 2.91; 95%CI: 1.32-6.44), while global obesity (BMI ≥ 35 Kg/m^2^) was highly predictive of abortion occurrence (OR = 2.49; 95%CI: 1.45-4.26). We observed no association between overweight women and fetal deaths.

**Table 3:** Adjusted OR (95% CI) of spontaneous abortions and stillbirths according to maternal anthropometry – Brazil, DHS/2006

**Maternal anthropometry**	**Spontaneous abortions**	**Stillbirths**	**Total deaths**
	**Adjusted**^**a **^**OR (95%CI)**	**Adjusted**^**a **^**OR (95%CI)**	**Adjusted**^**a **^**OR (95%CI)**
**Maternal BMI (Kg/m**^**2**^**)**			
Underweight (<18.5)	0.44 (0.19-1.02)	0.06 (0.01-0.48)^*^	0.37 (0.16-0.83)
Normal (18.5-24.9)	1.00	1.00	1.00
Overweight (25–29.9)	1.02 (0.64-1.64)	1.07 (0.43-2.67)	1.04 (0.68-1.59)
Obese 1 (≥ 30 -34.99)	1.14 (0.63-2.05)	2.16 (0.82-5.73)	1.29 (0.76-2.17)
Obese 2–3 (≥ 35)	2.49 (1.45-4.26)^*^	2.51 (0.46-13.74)	2.54 (1.49-4.31)^*^
**Maternal BMI (per 1 Kg/m**^**2**^**)**	1.05 (1.01-1.08)^*^	1.06 (1.00-1.13)	1.05 (1.02-1.08)^*^
**Maternal WC (cm)**			
Normal (< 80)	1.00	1.00	1.00
Overweight (80–87.9)	0.91 (0.58-1.43)	1.32 (0.48-3.67)	0.96 (0.64-1.46)
Obese (≥ 88)	1.30 (0.80-2.10)	2.91 (1.32-6.44)^*^	1.51 (1.00-2.31)
**Maternal WC (per 1 cm)**	1.02 (1.00-1.03)^*^	1.03 (1.00-1.06)	1.02 (1.01-1.04)^*^
**Maternal WHR**			
1st quartile	1.00	1.00	1.00
2nd quartile	0.98 (0.59-1.64)	0.89 (0.25-3.21)	0.97 (0.61-1.54)
3rd quartile	1.18 (0.73-1.92)	1.40 (0.48-4.12)	1.21 (0.78-1.89)
4th quartile	1.51 (0.89-2.59)	3.17 (1.17-8.56)^*^	1.75 (1.09-2.81)^*^
**Maternal WHR (per 0.1)**	1.32 (1.03-1.69)^*^	1.65 (1.06-2.57)^*^	1.39 (1.12-1.73)^*^

Notes: ^a^Adjusted for maternal age group, parity, skin color, household income, maternal education and maternal smoking status. *BMI* Body Mass Index, *WC* Waist Circumference, *WHR* Waist-to-Height Ratio, *OR* Odds Ratio, *SE* Standard Error, *95%CI* 95% Confidence Interval; ^*^p < 0.05.

### Sensitivity analysis

Furthermore, a sensitivity analysis (data not shown) was performed and consisted of evaluating all these relationships, but only among women who declared only one pregnancy. The results showed the same direction of the associations found for the entire population. Obese women with a BMI ≥ 35 Kg/m^2^ were found to have a greater chance of abortion, even after adjustment. Abdominally obese women had greater odds of stillbirth. Thus, for each unit increase in BMI, WC and WHR, the odds of total death were also increased. These associations remained significant even after adjusting for maternal age group, skin color, educational level, smoking status and household income, and confirmed the associations found for the whole population, regardless of the number of pregnancies.

## Discussion

### Main findings

In this study, obesity was associated with greater odds of spontaneous abortion and stillbirth among Brazilian women of reproductive age. This association was observed using different anthropometric markers of obesity (BMI, WC and WHR). The gain of each BMI unit increased the odds of spontaneous abortion and total deaths by approximately 5%. Women with higher BMI values (≥ 35.0 Kg/m^2^) had the highest chance of total death, and this obesity index was shown to be a more important predictor of abortion. Moreover, a WC ≥ 88 cm was a better predictor of the occurrence of stillbirths, with an almost 3-times greater chance compared with the baseline of optimal-waist circumference women (WC < 80 cm). WHR also presented a positive association with fetal outcomes. For each increase of 0.1 units of this ratio, women presented 32% and 65% increased odds of abortion and stillbirth, respectively. These results were adjusted for maternal age group, parity, skin color, household income, educational level and smoking status, which did not attenuate the associations found.

### Results in the context of other studies

These findings are consistent with those found in high-income countries [24-26] and low-income countries [12-14], although the majority of the studies focus on stillbirths and death after the child is born.

Anthropometric measures of obesity could contribute to fetal deaths. Similar studies have shown that obesity relates to the occurrence of adverse pregnancy outcomes, but most of these studies focused on maternal outcomes. We advanced these observations by considering fetal outcomes in the context of an emerging economy. Findings from a study that evaluated the risk of spontaneous abortion in 1,644 pregnant, obese women from a United Kingdom hospital facility compared to a control group of 3,288 women with normal BMI suggests an increased risk of abortion and recurrent early abortions in obese women [27].

Beyond population-based studies, obesity also impacts heavily on the clinical setting and the individual’s well-being. In addition to responding poorly to fertility treatments, obese women also have an increased risk of infertility [7]. Systematic reviews have shown that women who were overweight and obese had significantly lower clinical pregnancy and live birth rates (RR = 0.90 and 0.84), respectively. Obese women showed a higher abortion rate when compared to non-obese women (OR = 1.31, 95% CI 1.18 – 1.46) [28,29].

In addition to abortion, studies have also investigated the relationship of obesity leading to late fetal deaths. The results of one study that investigated the effect of obesity on pregnancy outcomes suggested that among nulliparous women in Sweden, the odds ratio for late fetal death was increased among women with a higher BMI compared with lean women (≤ 19.9 Kg/m^2^) [30]. This study also observed an increased risk among women with normal BMI levels (OR = 2.2; 95%CI: 1.2-4.1), overweight women (OR = 3.2; 95%CI: 1.6-6.2), and obese women (OR = 4.3; 95%CI: 2.0-9.3) [30]. Thus, among parous Swedish women, only obese women had a significant increase in the risk of late fetal death (OR = 2.0; 95%CI: 1.2-3.3) [30]. Similar results have been shown in Danish obese women, who presented a more than two-fold risk of stillbirth (OR = 2.8; 95% CI: 1.5-5.3) (BMI ≥ 30 Kg/m^2^) compared to normal BMI women [31]. Additionally, adjusted estimates showed a 63% greater likelihood of stillbirth among Swedish women who gained three or more BMI units between pregnancies when compared to those who changed less than one unit [24]. These authors also found that slight changes in BMI considerably increased the risk of adverse pregnancy outcomes. These studies corroborate our results, although we found WC to be related more to the occurrence of stillbirths than to BMI. Experimental data on obesity and abortion also provide support for our hypothesis [32,33].

### Limitations

The cross-sectional design of the DHS 2006 data limits our causal inference because it was difficult to determine temporality for the association of obesity and fetal deaths. To avoid the possibility of reverse causation, the ideal measurement should occur before pregnancy or during the first trimester. This absence of data is a serious limitation of our study, and only prospective primary data collection would resolve the issue. As previously reported, longitudinal data on this matter with nationally representative data are scarce, especially in low- and middle-income countries [26]. In an attempt to overcome this limitation, we performed an analysis with only primiparous women. This subset consisted of approximately a third of the entire study population and hindered possible weight gain after having several pregnancies. The results from this analysis remained unchanged and showed the same direction of the associations found in the analysis with the entire population, even after adjusting for the same confounders. We recognize that women could gain weight for some reasons other than multiple pregnancies, and this lack of temporal information indicates that our results should be interpreted carefully. Another way to overcome this limitation could be to evaluate whether changes in BMI after birth differed between women who lost their babies and those who did not [14]; however, because of the lack of exact data of the outcome (abortion or stillbirth), we could not perform this evaluation. Even so, our observations align in direction and magnitude with those previously reported in other high-income settings, indicating that this bias might not heavily affect our estimates. Additionally, there is also the possibility of residual confounding by unmeasured diseases and other conditions that may be associated with adverse pregnancy outcomes.

We should also note that the outcomes were self-reported. Although it is uncommon for a woman to forget such an event, directly measuring the occurrence of abortions and stillbirths would be the ideal assessment.

### Biological plausibility and implications

Despite these limitations, our findings are also consistent with those of similar studies, and plausible mechanisms that explain the link between obesity and fetal death have been proposed. This link may be directly related to obesity or to obesity-associated conditions, such as gestational diabetes and hypertensive disorders. Obesity is known to affect gonadal function, inhibiting ovarian follicle development throughout secreted adipokines that stimulate the hypothalamus. The adipokines also regulate embryo development in the early stages of cellular division, which, among other specific mechanisms, might justify the low reproduction performance in overweight and obese women [8]. Therefore, these changes could also explain harmful consequences to the child or even the unviability of the fetus. Additionally, previous studies have shown reproductive problems in obese women, such as proinflammatory cytokine accumulation, which could harm fetal membranes [34]; deregulation of metabolic, vascular and inflammatory pathways [10]; and hyperlipidemia, which reduces prostacyclin secretion and enhances peroxidase production, resulting in vasoconstriction and platelet aggregation [35]. We believe that some of these pathways offer plausible routes that provide reasonable explanations for the miscarriage and late fetal death observed in obese women.

The differential association of the two measures of obesity (BMI and WC) with early and late fetal deaths, respectively, is uncertain. This uncertainty might arise because WC is a more proximate measure of fat accumulation in the abdominal region, indicating a more deleterious form of obesity, a condition that is highly associated with inflammation, insulin resistance and the future development of diabetes [36]. Although very speculative, these events may have different triggers, and stillbirths are more closely related to a pre-diabetes condition while abortions are more closely related to the direct effects of obesity on reproductive function.

In public health terms, the findings of our study demonstrate the need for obstetric care providers to counsel obese reproductive-aged women regarding the risks, complications and importance of weight loss and weight control prior to pregnancy. In Brazil, non-communicable diseases have become the most important health problem. Brazil’s Unified Health System (SUS) provides primary health care based on Family Health Programme teams. Improvements in access to integral care towards the prevention of chronic diseases have been observed since its implementation and continuous expansion. Specific programs, such as smoking cessation, diabetes screening, and the distribution of low cost, generic and even free medications, have already been implemented. Despite these advances, critical aspects of the chronic care model of this program still remain, such as pressure for the incorporation of high complexity care rather than making good use of cost-effective technologies, and the absence of legislative and regulatory norms. Other health professionals, such as nutritionists, physical educators, psychologists, and psychiatrists, should support primary health care teams [37]. This initiative could promote physical activity and dietary modifications as a strategy to improve weight control and to avoid postpartum weight retention, consequently preventing maternal obesity. Thus, lifestyle changes could result in significant improvement in women’s reproductive function. From the standpoint of primary prevention, adverse pregnancy outcomes related to obesity may be preventable, and the identification of obesity in these women is a cost-effective prevention strategy because BMI calculation only requires simple measures, such as weight and height, along with the waist circumference, for the identification of abdominal obesity.

## Conclusions

In summary, we analyzed data from a large national demographic survey in Brazil with an emphasis on spontaneous abortions, stillbirths and total fetal death outcomes of participating women. Our results provide important epidemiological information in the direction of a potential positive relationship between markers of obesity and the risk of spontaneous abortion and stillbirth occurrence in a representative sample of the Brazilian population. Our study also used the WC and WHR ratio as an obesity index, and we believe that this use reinforces the importance of the results found. Moreover, our findings provide further justification for the development of effective strategies to reverse the trends toward a higher prevalence of maternal overweight and obesity.

## Abbreviations

95% CI: 95% Confidence interval; BMI: Body mass index; DHS: Demographic and health survey; OR: Odds ratio; RR: Risk ratio; SE: Standard error; WC: Waist circumference; WHR: Waist-to-height ratio.

## Competing interests

The authors declare that they have no competing interests.

## Authors’ contributions

MSFM participated in the conception, design, analysis and interpretation of data, and drafted the manuscript. FMP participated in the conception, design and data analysis, and helped to draft the manuscript. JJM helped to draft the manuscript and critically revised it for important intellectual content. EV participated in analysis and interpretation of data and critically revised it for important intellectual content. GVM conceived of the study, and participated in its design, interpretation of data, coordination, and critically revised it for important intellectual content. All authors read and approved the final manuscript.

## Pre-publication history

The pre-publication history for this paper can be accessed here:

http://www.biomedcentral.com/1471-2393/14/5/prepub
